# MR Product as a Novel Diagnostic Indicator for Chronic Secondary Mitral Regurgitation

**DOI:** 10.7759/cureus.10052

**Published:** 2020-08-26

**Authors:** Mishal Fatima, Nadir Mehmood, Syed Muhammad Jawad Zaidi, Muhammad Hamza, Mehwish Kaneez, Umer Irshad, Muhammad Junaid Azhar, Abdullah Bin Zubair, Rafay Rizwan, Muzammil Sabir

**Affiliations:** 1 Cardiology, Rawalpindi Medical University, Rawalpindi, PAK; 2 General Surgery, Rawalpindi Medical University, Rawalpindi, PAK; 3 Cardiology, Rashid Latif Medical College, Lahore, PAK

**Keywords:** left atrial diameter (lad), left ventricular internal dimension at end-systole (lvids), secondary mitral regurgitation (smr), mr product

## Abstract

Background

Chronic secondary mitral regurgitation (SMR) is a common form of valvular heart disease. Its diagnosis through echocardiography is challenging and dependent on subjective interpretations. The subjective error to diagnose SMR can be reduced by developing accurate predictive quantitative parameters that support echocardiographic interpretations and clinical manifestations. The present study aims to develop a new diagnostic indicator for chronic SMR. The new indicator called MR product is the product of left atrial diameter (LAD) and left ventricular internal dimension at end-systole (LVIDs).

Materials and Methods

An analytical, case-control study was conducted from transthoracic echocardiography (TTE) reports of 720 patients performed according to the guidelines of the American Society of Echocardiography. The LAD and LVIDs were measured using the standard M Mode TTE. Out of the 720 patients who underwent TTE, 300 patients were diagnosed with chronic SMR by experienced clinicians. Only 115 of those 300 patients met the inclusion criteria for chronic SMR.

Results

The MR product was significantly associated with chronic SMR (rho = 0.83) and predicted it with an odds ratio of 1.014 (p < 0.001). The MR product was able to diagnose SMR with a sensitivity of 94.8% and a specificity of 92.2%, respectively, for a cut off value of 1,045 mm^2^.

Conclusion

A new parameter called MR product (LAD multiplied with LVIDs) has very high sensitivity and specificity for SMR. Therefore, it can aid in establishing its diagnosis, along with other diagnostic modalities. The new parameter may also potentially increase the diagnostic accuracy of the disease.

## Introduction

Mitral regurgitation (MR) is a common form of valvular heart disease present in approximately 10% of adults over 75 years of age [1]. It is categorized based on the disease onset (acute or chronic) and the underlying pathology (primary or secondary). The underlying etiology of chronic secondary mitral regurgitation (SMR) involves functional valve abnormality due to ischemia or heart failure [2]. It is largely asymptomatic in the initial stages, and even in advanced cases (Grades 2 and 3). SMR may present with only mild symptoms, such as exercise intolerance and exertional dyspnea, which do not reflect the severity of the underlying disease process [1-2].

SMR is almost always associated with heart failure (HF) and one in every five patients with HF develops SMR [3]. About 20% of these patients have severe SMR, which increases the mortality risk by more than two times, while 80% of patients have a slow, progressive SMR, which is still an independent risk factor for increased mortality in HF patients [3-5]. The higher risk of mortality in such patients is caused by adverse cardiac remodeling due to the development of SMR [4]. Even without heart failure, SMR causes an increased risk of mortality [6]. These findings, combined with the fact the SMR remains largely asymptomatic until late stages, prompts the need for early diagnosis and management of such individuals [2, 7]. A potential area to improve these figures is to aid in establishing the diagnosis of chronic SMR with higher confidence so that early interventions can be performed to cure the pathology. 

The diagnosis of SMR usually begins with the detection of a heart murmur on routine cardiac examination of a heart failure patient who is typically in Grade 2 or 3 of SMR. Over the years, Doppler flow echocardiography has emerged as the standard method to diagnose a suspected case of SMR [7]. However, the echocardiographic diagnosis is not simple and requires identification of the pattern of associated valvular change, such as asymmetrical or symmetrical tenting, the measurement of coaptation distance (CD) and tenting area (TA), and gauging the degree of backflow of contrast jet, the jet flow area, and vena contracta width by echocardiography reports [8].

The accurate interpretation of a Doppler study requires a sound clinical acumen because it is dependent upon the clinician’s subjective assessment. Moreover, interpersonal differences between the interpretations may give rise to false diagnoses [9]. Hence, the dilemma of over or under-reporting of heart pathologies, especially those whose diagnosis is largely based on a physician’s subjective opinion on echocardiography reports (such as SMR), is a cause of concern in the medical community and guidelines for the standardization of the technique are regularly revised and published [9-10]. The routine use of Doppler studies further predisposes to subjective errors made by relatively inexperienced clinicians [11]. These diagnostic dilemmas are compounded by the fact that in developing countries, Doppler studies are not readily available in many areas. Therefore, a confirmatory diagnosis cannot be established in such cases [7].

The subjective error in diagnosing certain heart pathologies can be reduced by developing accurate predictive quantitative parameters that support echocardiographic interpretations so that the final diagnosis is not solely based upon the clinician’s opinion. One such parameter developed in a study by Haq et al. was the ratio of a left atrial to a left ventricular diastolic diameter which supports the diagnosis of Grade 2 diastolic dysfunction [12]. Thus, it is well-established that the development of quantitative parameters can potentially enhance the echocardiographic diagnosis of cardiac abnormalities.

The present study is concerned with the development of a new diagnostic indicator for chronic SMR. The new indicator is the product of the left atrial diameter (LAD) and the left ventricular internal dimension at end-systole (LVIDs). For the sake of simplicity, the product will be referred to as the MR product in the study. Increased LAD in SMR is well-established [13-14], but its low specificity prevents its use as a diagnostic indicator for heart pathologies. LVIDs are routinely measured in echocardiography and used for the assessment of left ventricular function, along with other ventricular parameters [15]. We could not find any literature that used LVIDs in assessing mitral regurgitation. In our study, the medical reason for using LVIDs in diagnosing secondary mitral regurgitation was the fact that SMR has been invariably associated with left ventricular dysfunction, and therefore, an increase in LVIDs can reflect the development of SMR.

The echocardiography machine measures both diameters of the MR product which minimizes the errors in subjective interpretation. The new indicator, when combined with other modalities, will help in establishing the diagnosis of this condition and may improve the diagnostic accuracy of chronic SMR. It will also help the clinicians to be more confident regarding their opinion on the echocardiography reports of such cases. Thus, the results of this study may add to the diagnostic arsenal for patients with SMR. The MR product can be measured by simple echocardiography (performed without contrast). If it is a valuable diagnostic indicator in our study, the MR product can be used as an initial diagnostic test for SMR, especially in those areas where contrast-based Doppler studies are not readily available.

## Materials and methods

Study design

An analytical, case-control study was conducted from March 2017 to September 2018 at the Department of Cardiology of the Holy Family Hospital, Rawalpindi, Pakistan. This study was approved by the Institutional Review Board of Rawalpindi Medical University (IRB-2016-MC-0420).

Echocardiography information

Transthoracic echocardiography (TTE) reports of 720 patients performed according to the guidelines of the American Society of Echocardiography were collected and documented [10]. The LAD and LVIDs were measured in millimeters (mm) using the standard M Mode TTE and documented. Reports with missing diameters or unclear images were excluded from the study.

Selection of cases

Three hundred of 720 patients (41.67%) were diagnosed with chronic SMR by experienced clinicians based on the report of the TTE Doppler echocardiography. We excluded patients with any autoimmune condition, malignancy, structural mitral valve disease (such as stenosis, prolapse, and rheumatic heart disease), mitral repair surgery or mitral valve implant, diastolic dysfunction of any grade, pericarditis, and sepsis from the study. Based on these criteria, 115 cases were then selected. 

Selection of controls

The patients who underwent Doppler echocardiography but did not have chronic SMR constituted our control population. The same exclusion criteria were applied and 115 controls were then selected after careful age and gender-based matching with the cases. 

Statistical techniques

The descriptive analysis of the study parameters was performed using the IBM Statistical Package for Social Sciences (SPSS), version 23.0 (IBM SPSS Statistics, Armonk, NY). Spearman’s correlation was used to observe the correlation between study variables. A Mann-Whitney U test was employed to assess significant differences in the study variables between cases and controls. A binary logistic regression was performed and the receiver operating characteristic (ROC) curve was constructed to evaluate the predictive power of the MR product for chronic SMR. A p-value of less than 0.05 was considered statistically significant.

## Results

Population parameters and their correlation with heart dimensions, indicated by Spearman’s correlation coefficient, are provided in Table 1. The table shows that the MR product has a stronger correlation (rho = 0.83) with chronic SMR than either LAD or LVIDs alone.

**Table 1:** Population Parameters and Their Correlation With Chronic SMR LAD: left atrial diameter; LVIDs: left ventricular internal dimension at end-systole; SMR: secondary mitral regurgitation

Parameters		Control group (n = 115)	Disease group (n = 115)	Spearman correlation rho value (with SMR)	p-value
Gender	Males	54	55	___	___
	Females	61	60		
Age		51.26 ± 18.13	54.13 ± 16.34	___	___
LAD (mm)		30.79 ± 4.41	39.55 ± 5.8	0.694	< 0.001
LVIDs (mm)		27.63 ± 4.16	37.98 ± 6.20	0.71	< 0.001
MR Product (LAD*LVIDs)		850.56 ± 171.49	1503.61 ± 338.78	0.833	< 0.001

Table 2 delineates the result of the Mann-Whitney U test. The mean rank was greater in SMR patients for LAD, LVIDs, and the MR product. This was highly significant (p < 0.001), indicating that larger values of the three parameters are significantly associated with SMR.

**Table 2:** Results of the Mann-Whitney U Test for Three Parameters LAD: left atrial diameter; LVIDs: left ventricular internal dimension at end-systole; SMR: secondary mitral regurgitation

Parameter	Mean Rank		p-value
	NO SMR	SMR	
LAD	69.30	161.30	< 0.001
LVIDs	66.30	164.70	< 0.001
MR Product (LAD*LVIDs)	60.22	170.78	< 0.001

Table 3 elucidates the results of binary logistic regression. The odds ratio was 1.014 which was highly significant (p < 0.001).

**Table 3:** Binary Logistic Regression Model for SMR CI: confidence interval; Exp B: exponentiation of the B coefficient (odds ratio); LAD: left atrial diameter; LVIDs: left ventricular internal dimension at end-systole

Variable	Regression Coefficient (B)	Odds Ratio (Exp B)	95% CI	p-value
MR product (LAD*LVIDs)	0.014	1.014	1.010 - 1.080	< 0.001

Table 4 shows that our binary logistic model correctly predicts 90.7% of cases as compared to 50% of cases of the null model.

**Table 4:** Predictive Capability of Binary Logistic Regression SMR: secondary mitral regurgitation

Observed		Predicted		
		Diagnosis		Percentage Correct
		No SMR	SMR	
Diagnosis	No SMR	109	6	94.8
	SMR	15	100	87.0
Overall percentage				90.9
The value of Nagelkerke R^2^ was 0.84				
The predictive capacity of the null model was 50.0%				

The receiver operating characteristic (ROC) curve characteristics of the three parameters (LAD, LVIDs, and MR product) are provided in Table 5. The area under the curve was highest for MR product at 0.981, showing a very high predictive power of the MR product for chronic SMR.

**Table 5:** Receiver Operating Characteristic (ROC) Curve of the Three Parameters CI: confidence interval; LAD: left atrial diameter; LVIDs: left ventricular internal dimension at end-systole

Parameter	Area	p-value	95% CI	Selected cut-off value	Sensitivity at the cut-off value	Specificity at the cut-off value
LAD	0.689	0.004	0.65 - 0.71	43.5 mm	75.7%	61.8%
LVIDs	0.754	< 0.001	0.72 - 0.78	38.5 mm	71.4%	69.5%
MR product (LAD*LVIDs)	0.981	< 0.001	0.97 - 0.99	1,045.00 mm^2^	94.8%	92.2%

## Discussion

Despite the heavy clinical burden of chronic secondary mitral regurgitation, its diagnosis and management offer many challenges, and long-term outcomes of surgical procedures have not been well-established, even in developed countries [16]. In developing countries, where contrast-based studies are readily accessible only at tertiary health care centers, the diagnosis rate of chronic SMR is even lower and many patients are diagnosed in the advanced irreversible stage associated with heart failure [7]. Although therapeutic interventions change the disease course of mitral regurgitation if instituted early, only a very few patients receive timely treatment. A study reported that only about half of the patients with primary MR received adequate therapeutic intervention [17]. While no statistics were found regarding the treatment ratio in SMR, it is presumed to be low as well. The lack of health education and moderate intensity of the symptoms add to the dismal picture surrounding this important cardiac pathology. Our study is an effort to aid in the diagnosis of chronic SMR, especially in areas where Doppler studies are not easily accessible, so that timely therapeutic interventions can be performed and better health care can be provided to these patients. 

The MR product employs two heart dimensions, LAD and LVIDs. Many studies have correlated LAD with chronic SMR and a few studies have established left atrial enlargement as a contributing factor for SMR [14]. Whether it’s a contributing factor for SMR or only an associated finding due to underlying left ventricular dysfunction is still yet to be determined. Enlargement of the left atrium is a routine finding on the echocardiography of SMR patients [14, 18]. Our results agree with these findings and the logical medical explanation is compensatory left atrial hypertrophy and dilatation in response to high-pressure backflow through the incompetent mitral valve.

When used alone as a diagnostic marker of SMR, the ROC curve of LAD shows sensitivity and specificity of 75.7% and 61.8%, respectively, at a cut off value of 43.5 mm, whereas the normal accepted left atrial diameter is 33.6 mm ± 4.1 [19]. The reason behind the low specificity and sensitivity is multifactorial causation of increased LAD, such as diastolic dysfunction, the normal aging process, hypertension, and obesity, which decrease its predictive accuracy for chronic SMR [20-21]. Therefore, although LAD is an independent risk factor for survival in SMR patients [22], its diagnostic utility is limited because of relatively low specificity. However, because of its clinical importance, LAD combined with other heart dimensions has been used as a marker for various heart pathologies [12]. It was employed as a ratio with left ventricular volume in a Chinese study for predicting hypertension-specific atrial enlargement [23]. Its role in predicting diastolic dysfunction was established in another study at Rawalpindi Institute of Cardiology [12]. Nevertheless, its role in the assessment of mitral regurgitation has not been studied. In our study, LVIDs shows a sensitivity and specificity of 71.4% and 69.5%, respectively, for chronic SMR, at a cut off value of 38.5 mm, whereas the normal accepted LVIDs is 29.9 ± 4.7 [19].

The new parameter uses the product of these two dimensions and shows a marked increase in predictive accuracy and strength of correlation than either LAD or LVIDs alone. The rho value of 0.83 indicates a very high strength of correlation with chronic SMR. At the area under the curve (AOC) of 0.98, the optimal sensitivity and specificity are 92.2% and 94.8%, respectively, for a cut off value of 1,045 mm^2^. This high predictive power is further validated by a binary logistic model which reports a predictive accuracy of 90.1% and Nagelkerke R^2^ of 0.84, meaning that our model, based on the MR product as a diagnostic indicator, is accurate in 90% of cases and explains 84% of the variability in the observations. The odds ratio of 1.014 was also highly significant (p < 0.001), indicating a strong relationship between the MR product and SMR. Thus, the statistics provide a strong logical basis for using the MR product as a diagnostic indicator of chronic SMR.

Based on these statistics, the MR product has the potential for many clinical applications in SMR. It may improve diagnostic accuracy when combined with other modalities, such as echocardiography and electrocardiography. It can be also be used for screening of suspected individuals before ordering a detailed contrast-based study, especially in areas where Doppler studies are expensive and not readily available. This application can increase the diagnostic rate and reduce health care burden by earlier diagnosis, timely interventions, and eventually, reduced mortality rates in such areas.

The study has certain limitations. Firstly, the diagnostic accuracy of the MR product needs to be confirmed by large clinical trials involving many geographic areas and it should be correlated with different patient parameters. Secondly, although the MR product may increase the diagnostic accuracy, its routine clinical use will require the proof of improved diagnostic rate of SMR by multiple studies.

## Conclusions

Chronic secondary mitral regurgitation (SMR) is a relatively prevalent condition with numerous diagnostic challenges and a poor clinical outcome when left undiagnosed and untreated. A new parameter called the MR product (LAD multiplied with LVIDs) has a very high sensitivity and specificity of 92.2% and 94.8%, respectively, for this condition. It can aid in establishing the diagnosis of SMR, along with other modalities, and may potentially increase the diagnostic accuracy for the disease. The MR product can also be used as a screening test for SMR in areas where contrast-based studies are not accessible on a large scale, thus ensuring better medical care for patients in these areas.
